# Stability of Executive Functioning of Moderately-Late Preterm and Full-Term Born Children at Ages 11 and 19: The TRAILS Cohort Study

**DOI:** 10.3390/ijerph18084161

**Published:** 2021-04-14

**Authors:** Sijmen A. Reijneveld, Jorijn Hornman, Sarai R. Boelema, Andrea F. de Winter

**Affiliations:** 1 Department of Health Sciences, University Medical Center Groningen, University of Groningen, 9700RB Groningen, The Netherlands; jorijn.hornman@gmail.com (J.H.); a.f.de.winter@umcg.nl (A.F.d.W.); 2 Siza Location’s Koonings Jaght, 6816TK Arnhem, The Netherlands; 3 Faculty of Social and Behavioral Science, Utrecht University, 3584CS Utrecht, The Netherlands; s.boelema@amsterdamumc.nl

**Keywords:** preterm birth, executive functioning, longitudinal, adolescence

## Abstract

Moderately-late preterm-born children (MLPs, 32–36 weeks gestational age, GA) have poorer executive functioning (EF) at primary school age than full-term children (FTs). Evidence is lacking on their EF in adolescence, but for early preterm-born children, this has been shown to be much poorer. We, therefore, compared EF of MLPs and FTs at ages 11 and 19 and assessed development between these ages. We obtained data from TRAILS, a community-based prospective cohort study in the northern Netherlands, on 98 MLPs and 1832 FTs. We assessed EF by the Amsterdam Neuropsychological Tasks (ANT) at ages 11 and 19 years and computed gender-specific z-scores on reaction time and accuracy. We compared baseline speed, pattern search, working memory, sustained attention, inhibition, and attentional flexibility of MLPs and FTs crude, and adjusted for small-for-GA status, socioeconomic status, and estimated intelligence. MLPs and FTs performed similarly on all EF components at ages 11 and 19, except for the speed, but not the accuracy measure of attentional flexibility. This was slightly poorer for MLPs than FTs at age 19 (adjusted B 0.25; 95% confidence interval: 0.00 to 0.50; *p* = 0.047), but not at age 11 (adjusted B −0.02; −0.19 to 0.22; *p* = 0.87). Differences in EF between MLPs and FTs did not change significantly from age 11 to 19. MLPs had comparable EF on most components as FTs, with only attentional flexibility at age 19 developing slightly poorer for MLPs than for FTs. These findings suggest the effects of MLP birth on long-term EF to be small.

## 1. Introduction

Birth below 37 weeks gestational age (GA) alters brain development [1,2], which may affect long-term outcomes [3,4]. The risks of altered brain development increase with the degree of prematurity [5], therefore most studies concerning the long-term outcomes of preterm children have focused on early preterm children (EPs, <32 weeks GA) [6,7]. However, most preterm born children (>80%) are moderately-late preterm born (MLP, 32–36 weeks GA) [8]. Long-term outcomes of MLPs have been shown to be more favorable than those of EPs, due to both their degree of prematurity and their risk of postnatal complications being lower. That lowers the risks of MLPs for brain damage and probably increases their potential for recovery later in life [5]. However, even if so, the overall community impact of the problems of MLPs may be larger as by far most preterm children are MLP [8,9]. Evidence on this is thus needed, but scarce.

Executive functioning is a core component of brain function and is essential for optimal cognitive and behavioral performance, as it concerns cognitive skills which mediate the ability to organize thoughts and behavior in a goal-directed manner [10,11]. Executive functioning can be divided into four domains, each consisting of different components: attentional control (such as sustained attention and inhibition), information processing (such as baseline speed), cognitive flexibility (such as working memory and attentional flexibility), and goal setting [12]. Preterm birth frequently affects the white matter integrity of the brain that underlies executive functioning, in particular in the prefrontal region, thalamus, and basal ganglia [12,13]. Contributing factors seem to be a disrupted process of myelination in preterm borns [13], and ischemic-hypoxic events that occur more frequently among preterm borns [7,14]. Poorer executive functioning may underlie the academic problems and the emotional and behavioral problems at a later age of MLPs [12].

MLPs have been shown to have poorer executive functioning between ages 3–8 years than full-term children (FTs) indeed [15,16,17], but recent evidence suggests that differences in executive functioning between MLP and FT do not persist into young adulthood [18]. However, evidence lacks on whether these executive problems still persist in adolescence. Adolescence is an important life stage that marks the transition from childhood to adulthood, with the maturation of cognitive functioning, especially executive functioning, being a major feature [11]. This transition regards several contexts of major importance for further life, i.e., from school to higher education and working, the initiation of intimate relationships, and the transition to independent living [19]. Studies on adolescent children born EP or with a very low birth weight (<1500 g) show that the executive functioning of these EPs is persistently poorer in all domains [12,20,21,22,23]. A recent meta-analysis of Van Houdt and coauthors shows that children born preterm/low birthweight performed worse on working memory, cognitive flexibility, and inhibition of 0.4, 0.4, and 0.5 standardized mean differences, respectively [7]. They found no significant diminishing of differences at higher ages (until about 14 years), and neither an apparent difference by gestational age. However, this meta-analysis retrieved very little data on MLPs, for this evidence is very scarce [7]. A study by Cserjesi and coauthors shows that at age 7, MLPs have a poorer executive functioning than full-terms [16]. Two recent studies suggest that this poorer executive functioning does not persist into adulthood [18,24]. Evidence on the age bracket in between, i.e., between age 7 and 18, lacks fully for MLP. Therefore, we aimed to compare the executive functioning of MLPs and FTs at age 11 and age 19 and to assess development between these ages. 

## 2. Materials and Methods

### 2.1. Participants

We used data from the first and fourth wave of Tracking Adolescents’ Individual Lives Survey (TRAILS); a prospective cohort study of children born between 1 October 1989 and 30 September 1990, who were included at age 11 years, i.e., the first wave. The study sample comprised children living in urban and rural areas of Northern Netherlands. Children were excluded (*n* = 215) if they had a severe psychical or mental handicap, language problems that made the completion of a questionnaire impossible, or a neurological tumor [11,25]. Of the eligible adolescents and their parents, 66% (*n* = 2230) agreed to participate and were enrolled in the first wave, at age 11 years [25]. A detailed overview of the participation rates and inclusion at ages 11 and 19 years can be found elsewhere [11,25]. 

**Figure 1:**
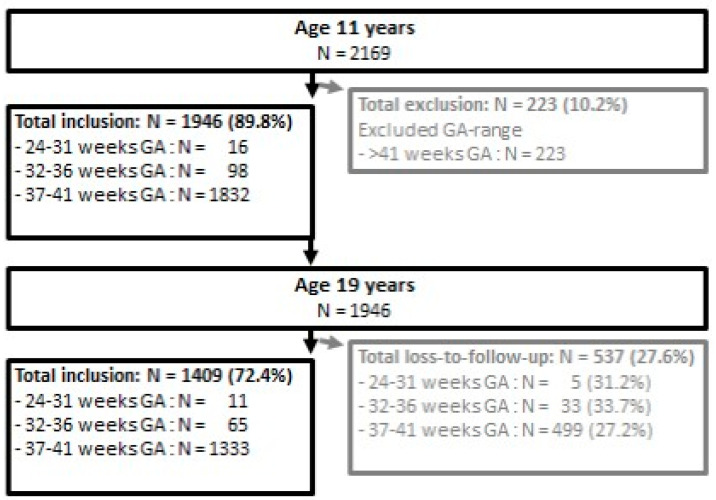
Flow chart of inclusion at age 11, and loss to follow-up between age 11 and 19, stratified by gestational age (GA).

Data on executive functioning were only collected in the first and fourth waves, i.e., at ages 11 and 19, and not in the second and third. At age 11, executive functioning data were available on 2169 children, from which were 239 (11%) outside the GA range of MLPs (32–36 weeks GA) and FTs (37–41 weeks GA). This resulted in 98 (5.1%) MLPs and 1832 FTs, of which we could retrieve at age 19: 65 MLPs and 1333 FTs (Figure 1). Loss to follow-up did not differ significantly between MLPs and FTs (33.7%. versus 27.2%; *p* = 0.165). The sample size at T1 (N1 = 98, N2 = 1832) provided a power of >80, at α = 0.05 to determine differences in z-scores of at least 0.30 SD (µ1 = 0, µ2 = 0.30, σ = 1) and the sample size at T4 (N1 = 65, N2 = 1333) provided a similar power to determine differences in z-scores of at least 0.36 SD (µ1 = 0, µ2 = 0.36, σ = 1).

The TRAILS study was approved by the Central Committee on Research Involving Human Subjects (Dutch CCMO). Parents’ and adolescents’ written informed consent was obtained.

### 2.2. Procedure and Measures

#### 2.2.1. Procedure

The executive functioning of the children at age 11 was assessed at their school or in designated testing centers by trained undergraduate psychology students [26]. At age 19, most adolescents were no longer in secondary education and were therefore tested individually by trained professional interviewers at home (24%) or a nearby community center (76%). The testers were trained in a workshop by researchers from the study on how to administer the computerized tasks. The workshop was followed by practice administrations on several test cases, which were monitored. At age 11, the completion time for the ANT was approximately 70 min (short breaks included). At age 19, time to complete the tasks took the respondents approximately 40 min on average; further details have been reported in Boelema et al., 2014 [11].

Furthermore, parents or guardians were interviewed and completed a questionnaire at home about perinatal aspects, family characteristics, and school problems at children’s age of 11. In addition, most parents (81.6%) also gave consent to use the reports of the child’s well-child visits, which gave us more detailed information about the perinatal characteristics. The testers of the Amsterdam Neuropsychological Tasks were not informed about the participants’ preterm birth and perinatal findings.

#### 2.2.2. Executive Functioning by the Amsterdam Neuropsychological Tasks

Executive functioning was assessed using the Amsterdam Neuropsychological Tasks (ANT) [27], at ages 11 and 19 years. The ANT has proven to be a sensitive and valid tool in both non-referred and referred samples [28,29,30]. We assessed information processing, attention control, and cognitive flexibility, divided into six components. The main outcome parameter of the six components was the computerized measured median reaction time and accuracy (percentage of errors per task). Each component and the outcome measurements are briefly described in Table 1; a detailed description can be found elsewhere [11,26]. We computed gender-specific FT-based z-scores for each component of the ANT separately at both age 11 and 19, to adjust for differences in executive functioning between boys and girls [11].

#### 2.2.3. Background Characteristics

We measured socioeconomic status, small for GA status and intelligence as background characteristics and as potential confounders because of their documented associations with both GA and executive functioning and not being on the causal path between these two [11,18,31]. Socioeconomic status was measured at child age 11 based on family income, level of education of the mother and the father, and level of occupation of the mother and of the father (using the International Standard Classification for Occupations [32]) [12]. These five variables were standardized each and next combined into one scale which had an internal consistency of 0.84 [12]. The lowest 25% of the scores were categorized as low socioeconomic status.

Small for gestational age was based on birthweight and GA derived from well-child visit reports, or if these lacked (18.4% of the participants) on parent-reports [33]. Small for gestational age (SGA) was defined as lighter than the tenth percentile of the Dutch Kloosterman gender-specific growth charts of intrauterine growth [34].

Intelligence was estimated based on child performance on the Vocabulary and Block Design subtests from the Revised Wechsler Intelligence Scales for Children (WISC-R) [35], at age 11. The scores on these subtests led to a WISC-Deviation Quotient score, based on the formula (15/standard deviation of sum score) × (sum score − 20) + 100 [36,37]. We further denote this as verbal and performance scores.

### 2.3. Statistical Analysis

First, we assessed the characteristics of the MLPs and FTs of the study sample at age 11 years and tested differences. Second, we compared scores on each ANT component for MLPs and FTs regarding reaction time and accuracy, using linear regression analyses. We did these regression analyses crudely and adjusted for the potential confounders, i.e., small-for-GA status, socioeconomic status, and verbal and performance score, and regarding the accuracy outcomes additionally adjusted for reaction time on the component concerned. Third, we assessed changes between age 11 and age 19 by repeating the multivariable analyses at age 19 with adjustment for their performance at age 11. All analyses were performed with IBM SPSS statistics version 25; results were considered significant with a *p* < 0.05.

## 3. Results

### 3.1. Background Characteristics

The characteristics of the MLPs and FTs at age 11 years are shown in Table 2. The MLPs had a significantly longer postnatal hospital stay than FTs. Verbal and performance scores and the rate of school problems were not different for MLPs and FTs.

### 3.2. Executive Functioning at Age 11 and Age 19

Table 3 and Table 4 show the reaction time and accuracy, respectively, of MLPs, compared with FTs on the Amsterdam Neuropsychological Tasks at ages 11 and 19. The z-scores in this table represent gender-specific FT-based z-scores, having a mean of 0.00 and a standard deviation of 1.00 in the FT group. A positive mean z-score for MLPs means a poorer performance for MLPs than for FT as it is a longer reaction time, a higher percentage of errors, or a larger difference in reaction time/accuracy between a simple and a difficult task. A negative mean score for MLPs is the opposite and thus a better performance for MLPs than for FTs (e.g., faster reaction time, fewer errors, etc.). The effect size beta is the regression coefficient resulting from crude linear regression analyses on z-scores. In the crude analyses, we found no significant difference in the executive functioning of MLPs and FTs. All outcome measures had variances that were equal for the MLP and FT group (Levene’s test *p* > 0.05), except for accuracy of working memory at age 11 and reaction time on attentional flexibility at age 19.

### 3.3. Change in Executive Functioning between Age 11 and Age 19

The adjusted analyses showed a significant difference between the reaction time of attentional flexibility of MLPs and FTs at age 19. However, there was no significant difference in attentional flexibility at age 11, the maturation between ages 11 and 19 in comparison with FTs, and neither on accuracy at both ages. We found no indications for multi-collinearity between the included independent variables in the adjusted analyses (VIF: prematurity 1.00, SGA 1.01, SES 1.04, and verbal and performance score 1.01).

## 4. Discussion

Our study showed that MLPs and FTs had a comparable executive functioning on most sub-functions at ages 11 and 19. MLPs only had poorer reaction time on attentional flexibility in comparison with FTs at age 19, but not at age 11. In addition, the accuracy of attentional flexibility and the maturation between age 11 and 19 was not significantly different between MLPs and FTs.

We found that MLP and FT adolescents had comparable executive functioning for most sub-functions at ages 11 and 19. Tideman et al. examined the cognitive development of 39 preterm children <35 weeks GA and 23 FTs at ages 4, 9, and 19 [38]. They found poorer cognitive development (on the Griffiths’ Total score) for preterm children in comparison with FTs at age 4, but a comparable cognitive development (including WAIS subtests and vasomotor speed (TMT test part A)) at later ages. Our results extend Tideman’s findings to full executive functioning in a larger study sample. They contrast however with findings on EPs which tend to have poorer executive functioning during this age period [12,20,22,39]. These contrasting findings may be due to their much stronger prematurity, and the increased risk of postnatal complications associated with a lower GA [40]. As a consequence, the risks of impaired white matter maturation and disturbing development of neuronal connections will be lower for MLPs than for Eps [5,41]. This leads to a better starting position for MLPs compared to EPs and may give them more potential for recovery. Consequently, MLPs may catch up before preadolescence [42], whereas executive problems persist in early preterm children until in adolescence. This shows that for MLPS the closing of the gap in executive functioning compared to FTs [18], may already occur in adolescence.

Another explanation for the comparability of the results for MLPs and FTs might be that our FT group also included children born at 37 weeks GA. There is increasing evidence that children who are born at 37 weeks have slightly poorer cognitive outcomes than children born between 38 and 41 weeks GA. Exclusion of children born at 37 weeks GA from the FT group did not affect our outcomes except that the difference in reaction times of attentional flexibility at age 19 between MLPs and FTs was no longer statistically significant. This finding strengthens our conclusion that MLPs have similar executive functioning as FTs in adolescence. A third explanation for the rather favorable outcomes of our MLPs may be that we have focused on more basic executive functioning. Therefore, we may not have detected subtler differences. Wehrle et al. showed that early preterm children with normal intellectual and motor function only have poorer executive functioning on more demanding levels and not on basic executive functioning at age 13 [23]. MLPs may thus still have a poorer performance on more subtle executive functioning tasks such as reasoning, problem-solving, and planning, but if so this can be expected to be much less disabling.

Looking at the mean reaction times on the attentional flexibility component, both MLPs and FTs showed maturation between ages 11 and 19, but FTs had a somewhat larger improvement than MLPs. Consequently, attentional flexibility at age 19 was significantly poorer in MLPs than in FTs. This finding may reflect either real differences between MLPs and FTs or be due to chance. Regarding the first interpretation, attentional flexibility is a subcomponent of cognitive flexibility, which has shown to be persistently poorer in early preterm adolescents in comparison with FT peers during adolescence [43,44,45]. The 19-year-old preterm adolescents <35 weeks GA (from the study by Tideman et al.) also showed poorer cognitive flexibility on the TMT part B test, but this was no longer statistically significant after adjustment for verbal and performance score and education of the mother [35]. The large maturation of attentional flexibility during adolescence is in line with our findings [11]. With the growing demands placed on the abilities of MLPs during adolescence, they may have increasing difficulties with more challenging executive tasks such as attentional flexibility in comparison with FTs [23,46]. This may for instance translate into being less able to participate in group discussions and to perform rapidly changing tasks in e.g., sports. Despite this seeming backlog, MLPs catch up sufficiently to reach a mostly similar executive functioning in young adulthood as FTs do [18]. Second, this may be a chance finding associated with the relatively many statistical comparisons that we made. This evidently requires further study.

The strengths of this study are the large community-based cohort with repeated extensive measures of executive functioning. Furthermore, we could correct for major confounders such as socioeconomic status, small for GA, and verbal and performance score. A limitation of this study is the relatively small number of MLPs, which may have left some associations unnoted, though our sample size allows us to detect the most clinically relevant differences. Moreover, we had a loss to follow-up of about 30% which could have biased findings. However, this loss was rather similar for MLPs and FTs, making this bias less likely.

Our findings suggest a rather favorable prognosis of executive functioning in MLPs. In contrast to EPs [12,20,21,22,23], MLPs seem to catch up somewhere between ages 8 and 11 [15,16,17], leading to a largely comparable executive functioning for MLPs and FTs at ages 11 and 19. This implies a relatively favorable long-term prognosis for MLPs with most problems disappearing in adolescence and not persisting to later life [18], increasing the opportunities for MLP in education and entry to the labor market. These findings need confirmation as this regards one of the first studies that was community-based and assessed executive functioning of MLPs in adolescence, both regarding executive functioning and regarding educational and labor market chances. Such a confirmatory study should preferably also include other GA categories as a comparison.

## 5. Conclusions

Long-term executive functioning outcomes were similar for MLPs and FTs, however, our results suggest poorer attentional flexibility at age 19 among MLPs. These findings are hopeful and suggest that they are at average poorer executive functioning already improves in adolescence. This definitely needs confirmation given the impact on policies in public health and neonatal follow-up.

## Figures and Tables

**Table 1:** Description of the outcome measures reaction time and accuracy on six components of the Amsterdam Neuropsychological Tasks.

**Domain Information Processing**
**Component 1 Baseline Speed: Simple visuomotor time.**
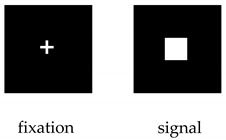
*Task*: The task consists of one part with the left and one with the right index finger starting with the nondominant index finger in the first part. Each part consists of 32 trials. On the computer screen, a cross is depicted which changes, at unexpected moments, into a square, see figure. When the participant sees the square s/he has to directly press the mouse button with the index finger. Cognition is limited to the detection of the mere presence of the signal. *Outcome reaction time*: time to detect and respond to a stimulus. A shorter reaction time indicates a better performance.
**Domain Attention Control**
**Component 2 Sustained Attention**
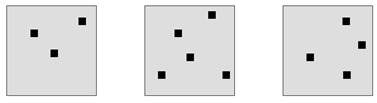
*Task*: The participant is shown 600 pictures with 3, 4, or 5 dots (200 trials of each type of stimulus, see figure). The target signal is the one with the 4 dots, and the participant has to indicate whether this target signal is shown in the picture by pressing the mouse button with the dominant index finger ("yes") or non-dominant index finger ("no"). The participant hears a sound when he/she makes a mistake. The primary sustained attention index is fluctuation in tempo. *Outcome reaction time*: Within-subject SD per set of 50 trials. A smaller SD indicates a better performance. *Outcome accuracy*: overall % of errors on the set of 50 trials. A lower % indicates a better performance.
**Component 3 Inhibition: Inhibition of prepotent responses.**
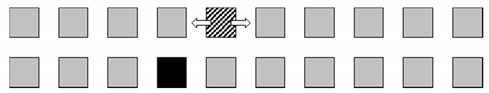
*Task*: A square jumping randomly left/right on a horizontal bar (containing 10 grey squares). The task consists of two parts, each consisting of 40 trials. In the first part, one of the ten squares is green and jumping randomly left/right on the horizontal bar, see figure, upper row. If the green square jumps left, the participant has to press the left mouse button and the right mouse button if it jumps right; this is the fixed compatible response condition. In the second part, one of the ten squares is red and jumping randomly left/right on the horizontal bar, see figure, lower row. If the red square jumps left, the participant has to press the right mouse button and vice versa; this is the fixed incompatible response condition. *Outcome reaction time*: Subtracting reaction time of correct responses in the fixed compatible response condition from reaction time of correct responses in the fixed incompatible response condition. A smaller difference is better. *Outcome accuracy:* Subtracting % of errors on fixed compatible response condition from % of errors on fixed incompatible response condition. A smaller difference is better.
**Domain Cognitive Flexibility**
**Component 4 Pattern Search: Automatic and controlled visuospatial pattern recognition.**
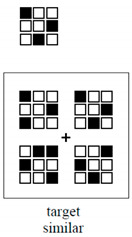
*Task*: A visuospatial target pattern is presented of 9 blocks in a 3 × 3 matrix. From the 9 blocks, 3 are red and 6 are white-colored, which are ordered in a certain way. In this task, 4 patterns of 3 × 3 matrixes are presented. In half of the signals of the task, the target pattern is one of the 4 presented patterns; this is the target condition. In the other half, the target pattern is not part of the 4 presented patterns; this is the non-target condition. The participant should press "yes" in the target condition and "no" in the non-target condition. In half of the signals, the target pattern is hard to differentiate from the other presented patterns, and in the other half, the target pattern is easy to differentiate from the other patterns. *Outcome reaction time*: Difference in reaction time needed to correctly identify non-similar patterns which are hard to differentiate and which are easy to differentiate. A smaller difference indicates a better performance. *Outcome accuracy:* Difference in % of false alarms to identify non-similar patterns which are hard to differentiate and which are easy to differentiate. A smaller difference indicates a better performance.
**Component 5 Working Memory: Working memory capacity**
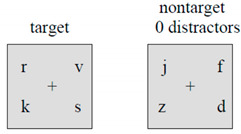
*Task*: The task comprises three parts, and each part depicts pictures with four letters, see figure for two examples. In the first part, consisting of 40 trials, participants have to indicate whether the letter "k" is present in the picture by pressing the mouse button with the either dominant index finger ("yes") or the non-dominant index finger ("no"). In the second part consisting of 72 trials, participants have to indicate whether both letters "k" and "r" are present in the picture. In the third part consisting of 96 trials, they have to indicate whether all three letters ("k," "r," and "s") are shown in the picture. Half of the trials in each part contain a target. This task provides an index for memory search capacity (deterioration in speed as a function of memory load). *Outcome reaction time*: Difference in reaction time of correct responses between the setting of a high working memory load task and a low working memory load task. A smaller difference in reaction time indicates a better performance. *Outcome accuracy:* Difference in % of errors in the setting of a high working memory load task and a low working memory load task. A smaller difference indicates a better performance. **Component 6 Attentional Flexibility** *Task*: The first part is the fixed compatible response condition as described in the inhibition task, see that figure. The second part is a combination of the fixed compatible response condition and the fixed incompatible response condition (as described for the outcome inhibition). The square will randomly jump right/left and will turn green/red. When the square is green after the jump, the participant has to press the button in the same direction while if the square becomes red after the jump the participant has to press the opposite button; changing condition. *Outcome reaction time*: Subtracting reaction time of correct responses in fixed compatible response condition from reaction time of correct responses in the changing condition. A smaller difference indicates a better performance. *Outcome accuracy:* Subtracting % or errors in fixed compatible response condition from % of errors in the changing condition. A smaller difference indicates a better performance.

**Table 2:** Characteristics of the study sample by gestational age category (full-term vs. moderately-late preterm), at age 11 years.

	Full-Term	Moderately-Late Preterm	
	N (%)/Mean (SD)	N (%)/Mean (SD)	*p* *
Participants at age 11 years	1832	98	
Participants at age 19 years (% of age 11)	1333 (72.8)	65 (66.3)	0.165
Male	904 (49.3)	45 (45.9)	0.509
Gestational age (weeks)	39.69 (1.05)	34.87 (1.42)	<0.001
Low socioeconomic status	461 (25.2)	22 (22.4)	0.546
Ethnicity-Dutch	1583 (86.4)	88 (89.8)	0.789
Moroccan/Turkish	23 (1.3)	0 (0.0)	
Other	227 (12.4)	10 (10.2)	
Birth weight (grams)	3435 (490)	2435 (605)	<0.001
Small for gestational age <10th percentile	245 (13.4)	19 (19.4)	0.091
Postnatal days in hospital	4.05 (8.4)	15.27 (14.6)	<0.001
School problems ^#^	402 (21.9)	25 (25.5)	0.407
Verbal and performance score, based on:	97.39 (14.92)	97.39 (15.02)	0.997
WISC-R vocabulary test (vocabulary score)	9.09 (2.84)	9.49 (2.81)	0.172
WISC-R block design test (spatial score)	10.05 (3.10)	9.64 (2.97)	0.206
Median age time point 11 years (N = 1946)	11.10 (0.55)	11.07 (0.54)	0.628
Median age time point 19 years (N = 1409)	19.19 (0.57)	19.11 (0.53)	0.295

* *p*-values were assessed with Chi-square tests, unpaired T-tests, and Mann–Whitney U tests. ^#^ repeated a grade or special education at primary school (till age 11/12).

**Table 3:** Associations between gestational age and executive functioning regarding reaction time. Comparison of the mean reaction times on the Amsterdam Neuropsychological Tasks at ages 11 and 19 for MLP and FT children, differences in (gender-specific full-term born based) z-scores, and results of the crude and adjusted linear regression analyses on z-scores; the regression coefficient ‘effect size beta’ (based on crude analyses); and the crude and adjusted *p*-values.

Measures	RT FT Mean (SD)	RT MLP Mean (SD)	RT Z-Score MLP Mean (SD)	Effect Size Beta (95% CI)	*p* Crude	*p* Adjusted *	*p* Adjusted for Age 11 ^#^
**Age 11 years**							
Baseline Speed	309 (39)	307 (40)	−0.10 (0.95)	−0.09 (−0.29 to 0.12)	0.39	0.37	
Pattern search	1469 (485)	1523 (534)	0.11 (1.09)	0.11 (−0.10 to 0.31)	0.31	0.28	
Working memory	470 (259)	488 (268)	0.10 (1.06)	0.09 (−0.11 to 0.30)	0.38	0.37	
Sustained attention	1.73 (0.90)	1.89 (0.94)	0.18 (1.07)	0.19 (−0.02 to 0.39)	0.07	0.06	
Inhibition	199 (161)	185 (136)	−0.9 (0.89)	−0.08 (−0.28 to 0.12)	0.43	0.45	
Attentional flexibility	557 (221)	562 (205)	−0.03 (0.91)	0.02 (−0.19 to 0.22)	0.88	0.87	
**Age 19 years**							
Baseline Speed	237 (22)	235 (19)		−0.06 (−0.31 to 0.19)	0.65	0.76	0.94
Pattern search	815 (269)	829 (286)	0.05 (1.05)	0.05 (−0.20 to 0.30)	0.70	0.43	0.44
Working memory	236 (147)	231 (142)	−0.02 (0.94)	−0.02 (−0.27 to 0.23)	0.86	0.92	0.81
Sustained attention	0.88 (0.45)	0.91 (0.41)	0.13 (1.01)	0.09 (−0.16 to 0.34)	0.47	0.22	0.99
Inhibition	169 (141)	147 (128)	−0.13 (0.96)	−0.16 (−0.41 to 0.09)	0.20	0.34	0.24
Attentional flexibility	337 (142)	371 (187)	0.15 (1.18)	0.22 (−0.03 to 0.48)	0.09	0.047	0.07

* *p* adjusted: Adjusted for being small for gestational age, having a low socioeconomic status, and verbal and performance score ^#^ *p* adjusted for age 11: Additionally adjusted for the sub-function at age 11. RT = reaction time; SD = standard deviation; 95% CI = 95% confidence interval.

**Table 4:** Associations between gestational age and executive functioning regarding accuracy. Comparison of the percentages of errors on the Amsterdam Neuropsychological Tasks at ages 11 and 19 for MLP and FT children, differences in (gender-specific full-term born based) z-scores, and results of the crude and adjusted linear regression analyses on z-scores; the regression coefficient ‘effect size beta’ (based on crude analyses); and the crude and adjusted *p*-values.

Measures	% Errors FT Mean (SD)	% Errors MLP Mean (SD)	% Errors z-Score MLP Mean (SD)	Effect Size Beta (95% CI)	*p* Crude	*p* Adjusted *	*p* Adjusted for Age 11 ^#^
**Age 11 years**							
Pattern search	3.78 (9.06)	3.37 (7.28)	−0.05 (0.79)	−0.05 (−0.25 to 0.15)	0.649	0.653	
Working memory	−3.36 (7.78)	−3.05 (5.90)	0.03 (0.75)	0.04 (−0.16 to 0.24)	0.714	0.710	
Sustained attention	5.09 (3.15)	4.61 (2.98)	−0.14 (0.98)	−0.14 (−0.35 to 0.06)	0.172	0.111	
Inhibition	5.88 (9.03)	5.89 (7.79)	0.00 (0.86)	0.00 (−0.20 to 0.20)	0.988	0.817	
Attentional flexibility	9.01 (13.01)	9.92 (15.01)	0.06 (1.19)	0.06 (−0.14 to 0.27)	0.553	0.841	
**Age 19 years**							
Pattern search	2.92 (9.48)	1.77 (5.41)	−0.11 (0.60)	−0.12 (−0.36 to 0.13)	0.917	0.462	0.463
Working memory	−4.05 (7.35)	−3.83 (6.98)	0.02 (0.95)	0.13 (−0.22 to 0.27)	0.843	0.818	0.853
Sustained attention	4.17 (3.66)	3.83 (2.28)	−0.06 (0.78)	−0.05 (−0.30 to 0.19)	0.671	0.677	0.846
Inhibition	5.51 (9.76)	5.92 (10.12)	0.04 (1.07)	0.04 (−0.21 to 0.29)	0.756	0.373	0.579
Attentional flexibility	1.63 (5.67)	2.21 (7.57)	0.06 (1.23)	0.06 (-0.19 to 0.32)	0.622	0.692	0.419

* *p* adjusted: Adjusted for reaction time, being small for gestational age, having a low socioeconomic status, and verbal and performance score ^#^ *p* adjusted for age 11: Additionally adjusted for the sub-function at age 11 SD = standard deviation; 95% CI = 95% confidence interval.

## Data Availability

The data that support the findings of this study are available from the TRAILS consortium (www.trails.nl; accessed on 14 April 2021), and also stored a DANS EASY (https://easy.dans.knaw.nl; accessed on 14 April 2021). Access can be obtained from the authors upon reasonable request and with permission of the TRAILS consortium.
